# Landscape of somatic mutations in breast cancer: new opportunities for targeted therapies in Saudi Arabian patients

**DOI:** 10.18632/oncotarget.27909

**Published:** 2021-03-30

**Authors:** Duna H. Barakeh, Rasha Aljelaify, Yara Bashawri, Amal Almutairi, Fatimah Alqubaishi, Mohammed Alnamnakani, Latifa Almubarak, Abdulrahman Al Naeem, Fatema Almushawah, May Alrashed, Malak Abedalthagafi

**Affiliations:** ^1^Department of Pathology, King Saud University Medical City, Riyadh, Kingdom of Saudi Arabia; ^2^Genomics Research Department, Saudi Human Genome Project, King Fahad Medical City and King Abdulaziz City for Science and Technology, Riyadh, Kingdom of Saudi Arabia; ^3^Department of Biostatistics, Research Centre, King Fahad Medical City, Riyadh, Kingdom of Saudi Arabia; ^4^Department of Pathology, King Fahad Medical City, Riyadh, Kingdom of Saudi Arabia; ^5^Department of Radiology, King Fahad Medical City, Riyadh, Kingdom of Saudi Arabia; ^6^Department of Surgery, King Fahad Medical City, Riyadh, Kingdom of Saudi Arabia; ^7^Department of Clinical Laboratory Sciences, College of Applied Medical Sciences, King Saud University, Riyadh, Kingdom of Saudi Arabia; ^8^Chair of Medical and Molecular Genetics Research, King Saud University, Riyadh, Kingdom of Saudi Arabia

**Keywords:** breast cancer, PIK3CA, BCa, Saudi Arabia, BRCA

## Abstract

Breast cancer (BCa) ranks first in incidence rate among cancers in Arab females. The association between genetic polymorphisms in tumor suppressor genes and the risk of BCa has been studied in many ethnic populations with conflicting conclusions while Arab females and Saudi Arabian studies are still lacking. We screened a cohort of Saudi BCa patients by NGS using a bespoke gene panel to clarify the genetic landscape of this population, correlating and assessing genetic findings with clinical outcomes. We identified a total of 263 mutations spanning 51 genes, including several frequently mutated. Among the genes analyzed, the highest mutation rates were found in PIK3CA (12.9%), BRCA2 (11.7%), BRCA1 (10.2%), TP53 (6.0%), MSH2 (3.8%), PMS2 (3.8%), BARD1 (3.8%), MLH1 (3.4%), CDH1 (3.0%), RAD50 (3.0%), MSH6 (3.0%), NF1 (2.6%), in addition to others. We identified multiple common recurrent variants and previously reported mutations. We also identified 46 novel variants in 22 genes that were predicted to have a pathogenic effect. Survival analysis according to the four most common mutations (BRCA1, BRCA2, TP53, and PIK3CA) showed reduced survival in BRCA1 and BRCA2-mutant patients compared to total patients. Moreover, BRCA2 was demonstrated as an independent predictor of reduced survival using independent Cox proportional hazard models.

We reveal the landscape of the mutations associated with BCa in Saudi women, highlighting the importance of routine genetic sequencing in implementation of precision therapies in KSA.

## INTRODUCTION

Worldwide, breast cancer is the most common cancer in women. Arab and Middle-Eastern women have a high risk of cancer with an average age at diagnosis of 48 years, which is almost ten years earlier than in western countries [1]. According to estimates from the GLOBOCAN 2018 database, BCa ranks second in terms of cancer incidence and is the fourth leading cause of cancer-related mortality worldwide. Approximately 2.1 million new BCa diagnoses were reported globally by GLOBOCAN in 2018, comprising 11.6% of all new cancer diagnoses [2].

The heterogeneous nature of BCa has led to the development of a classification system based on expression profiles. Genome-wide RNA expression profiling subdivides BCa into five classes according to gene expression profiles. The classes are based on the expression of immunohistochemical (IHC) tissue markers as indicated by: (1) estrogen receptor (ER) positivity; (2) progesterone receptor (PR) positivity; (3) human epidermal growth factor receptor 2 (HER2) positivity; (4) proliferation index (marked by the Ki67 protein); and (5) the expression of a unique cluster of genes termed the basal cluster (TNBC) [3, 4]. The expression patterns of these genes define the molecular signature for each subtype. Accordingly, positive ER and/or PR, negative HER2, and low levels of Ki67 suggest the luminal A BCa subtype, which is the most common and displays the best prognosis. A positive ER and/or PR, either positive HER2 or negative HER2, and high levels of Ki67 suggest a luminal B BCa subtype, which makes up less than 20% of all BCa cases and has lower survival rates than luminal A. The absence of ER and PR expression accompanied by high expression of HER2 and proliferation gene clusters and low expression of luminal and basal clusters, as detected by IHC, suggests a HER2-enriched BCa subtype, which accounts for 10% to 15% of all cases and has a poorer prognosis than luminal cancers. Negative ER, negative PR, and negative HER2 suggest a triple-negative/basal-like BCa (TNBC) subtype, which makes up 20% of all BCas. This subtype is aggressive and manifests at earlier ages [5–9].

Approximately 10–20% of BCa patients have at least one affected first-degree relative. Up to 20% carry germline mutations in the BCa susceptibility tumor suppressor genes 1 or 2 (BRCA1 or BRCA2). The majority of these mutations are frameshifts that generate premature stop codons and decrease the production of a functional BRCA protein [1]. BRCAs are tumor suppressors that play an important role in DNA damage repair through homology-directed repair (HDR). Mutations in genes other than BRCA tumor suppressors account for less than 1% of all inherited BCas [10–13]. For instance, ATM mutations are responsible for the development of ataxia telangiectasia (AT). AT patients have a significant potential to develop BCa by the age of 50. The ATM gene is involved in DNA damage repair [14]. Similarly, PALB2 (an interacting partner of BRCA1 and BRCA2 and CHEK2) is known to carry loss-of-function mutations implicated in hereditary BCa [15].

Numerous signaling pathways involved in healthy development have been implicated in BCa progression. These pathways are often linked to cell proliferation, apoptosis, differentiation, and motility [16]. Three significant pathways govern mammary gland and BCa stem cell development: (1) estrogen receptor (ER) signaling; (2) HER2 signaling; and (3) canonical Wnt signaling. In ER signaling, estrogen binds membrane estrogen receptors and triggers a cascade of events that ultimately promote the binding of nuclear estrogen receptors (ERα, ERβ) with estrogen response elements (EREs). BRCA1 acts as a corepressor and inhibits ERα signaling [17], while cyclin D1 binds to ERα and supports BCa growth [3]. In HER2 signaling, human epidermal growth factor receptor-2 (HER-2) dimerizes as a result of ligand binding. This leads to the phosphorylation of tyrosine residues in the intracellular domain of HER2 and the activation of downstream pathways, including the mitogen-activated protein kinase (MAPK) and phosphatidylinositol 4,5-bisphosphate 3-kinase (PI3K) pathways [18]. Similarly, binding of the Wnt–receptor to its ligand activates canonical Wnt/β-catenin, which subsequently leads to the regulation of oncogenic gene expression, including MYC, CCND1, MMP7 and CD44 [19]. Other pathways involved in BCa development include cyclin-dependent kinase (CDK) signaling, notch signaling, sonic hedgehog (SHH) signaling, breast tumor kinase (BRK) signaling, and PI3K/AKT/mTOR signaling [20–23].

Many genes that are susceptible to oncogenic mutations are linked to BCa development. Somatic mutations in PIK3CA account for approximately 30% of the processes that enhance PI3K/AKT/mTOR signaling, the most common oncogenic signaling pathway linked to BCa [24]. In general, PIK3CA mutations are useful prognostic markers and are prevalent in ER-positive/HER2-negative tumors; there is also new evidence of PIK3CA mutation prevalence in HER2-positive tumors [25, 26]. The majority of PIK3CA somatic mutations cluster at two hot spots, one in exon 9 (E542K or E545K) and the other in exon 20 (H1047R or H1047L) [27]. Mutations in PIK3R1 are also implicated in BCa, albeit with lower frequencies. Other PI3K-enhancing mechanisms, such as the amplification of HER2, the loss of PTEN function, and the introduction of AKT1 activating mutations, have also been reported [28]. Somatic mutations in TP53 are also frequent in a large number of human BCas [29].

A genomic model for BCa was generated using next-generation sequencing (NGS). Genome-wide association studies (GWAS) have identified various BCa-associated loci. Five risk loci have been reported since 2007 using GWAS, with approximately 1000 loci still unidentified [30]. Two other loci were found to be associated with BCa in African women in 2013, [31] and three were found to be associated with BCa in Asian women in 2014 [32]. Novel mutations in BRCA1, BRCA2, and PALB2 were also identified in breast and ovarian cancer using whole genome amplification (WGA) [33]. Whole-exome sequencing (WES) was used to detect rare deletions in BRCA2 linked to male BCa risk, [34] and rare mutations in FANCC and BLM were identified as susceptibility alleles for BCa [35]. WES also facilitated both the identification of the FANCM gene as a susceptible gene for triple-negative BCa [36] and the association of XCR1, DLL1, TH, ACCS, SPPL3, CCNF and SRL with BCa. Unlike GWAS and WES, targeted sequencing addresses known loci, allowing accelerated mutation detection rates and accurately targeted therapy [37]. Targeted therapies for BCa are used to treat patients who overexpress ER, HER1, HER2, and vascular endothelial growth factor (VEGF). Directed therapy includes inhibitors of PI3K/AKT/mTOR, RAS/MEK/ERK, SRC kinase, insulin-like-growth-factor [IGF/IGF-receptor (IGFR)], poly-ADP ribose polymerase (PARP), and matrix metalloproteases (MMPs) [38].

According to the 2014 Saudi Cancer Registry (SCR), BCa is the most prevalent cancer in Saudi women (approximately 28.7% of all cancers). Approximately 78% of Saudi BCas are the IDC type. Although Saudi Arabia has a lower age-standardized rate (ASR) for female BCa than Western countries, a stable increase in the incidence of BCa has been observed, specifically in the Eastern Province [39]. At the molecular level, the most common BCa subtypes in the Saudi population include luminal A (58.5%), triple-negative (14.8%), luminal B (14.5%), and HER2-positive (12.3%) [40]. Clinically, these figures represent a robust diagnostic measure that can direct personalized therapy. In this study, we screened a cohort of Saudi BCa patients using a cancer-specific gene panel to ascertain the mutation spectrum and explore the possible clinical implications of the identified somatic variants in BCa development.

## RESULTS

Fifty-three cases were sequenced. Of the cases, 20 samples (37.7%) were luminal, 13 samples (24.5%) were TNBC, 7 samples (13.2%) were HER2-enriched and 13 samples (24.5%) were not classified (Table 1). Fifty-one samples (96%) were IDC, one was diagnosed as IDC with atypical medullary features and one as IDC with micropapillary features. Four samples had a metastatic disease corresponding to stage IV (7.5%), while remaining samples (92%) presented with a localized disease (Supplementary Table 1). Charts analysis revealed that all excision samples have received neoadjuvant therapy (49 patients, 92%). Adjuvant chemotherapy administered was 3 cycles of FEC100 (5 fluorouracil, epirubicin and cyclophosphamide) and Docetaxa, while patients with luminal tumors received a regimen of Tamoxifen. Adjuvant Radiotherapy was also administered.

**Table 1:** Patient clinical characteristics summary

Characteristic	Total number
Total (%)	53 (100%)
Age Average (range)	52.2 (32–76)
Gender	Female
Special Histopathology Subtypes	• IDC with atypical medullary cancer features (*n* = 1). • IDC with micropapillary features (*n* = 1)
SBR^*^ GRADE	
I	2 (3.7%)
II	28 (52.8%)
III	23 (43.3%)
DCIS	30 (56.6%)
HORMONE MARKERS	
ER/PR (Luminal)	20 (37.7%)
HER2-NEU	7 (13.2%)
TNBC	13 (24.5%)
Unclassified	13 (24.5%)
COMMON GENES	
BRCA1	16 (30.18%)
BRCA2	20 (37.7%)
TP53	14 (26.4%)
PIK3CA	30 (56.6%)

^*^SBR: Nottingham grading system

### Mutations

**Figure 1:**
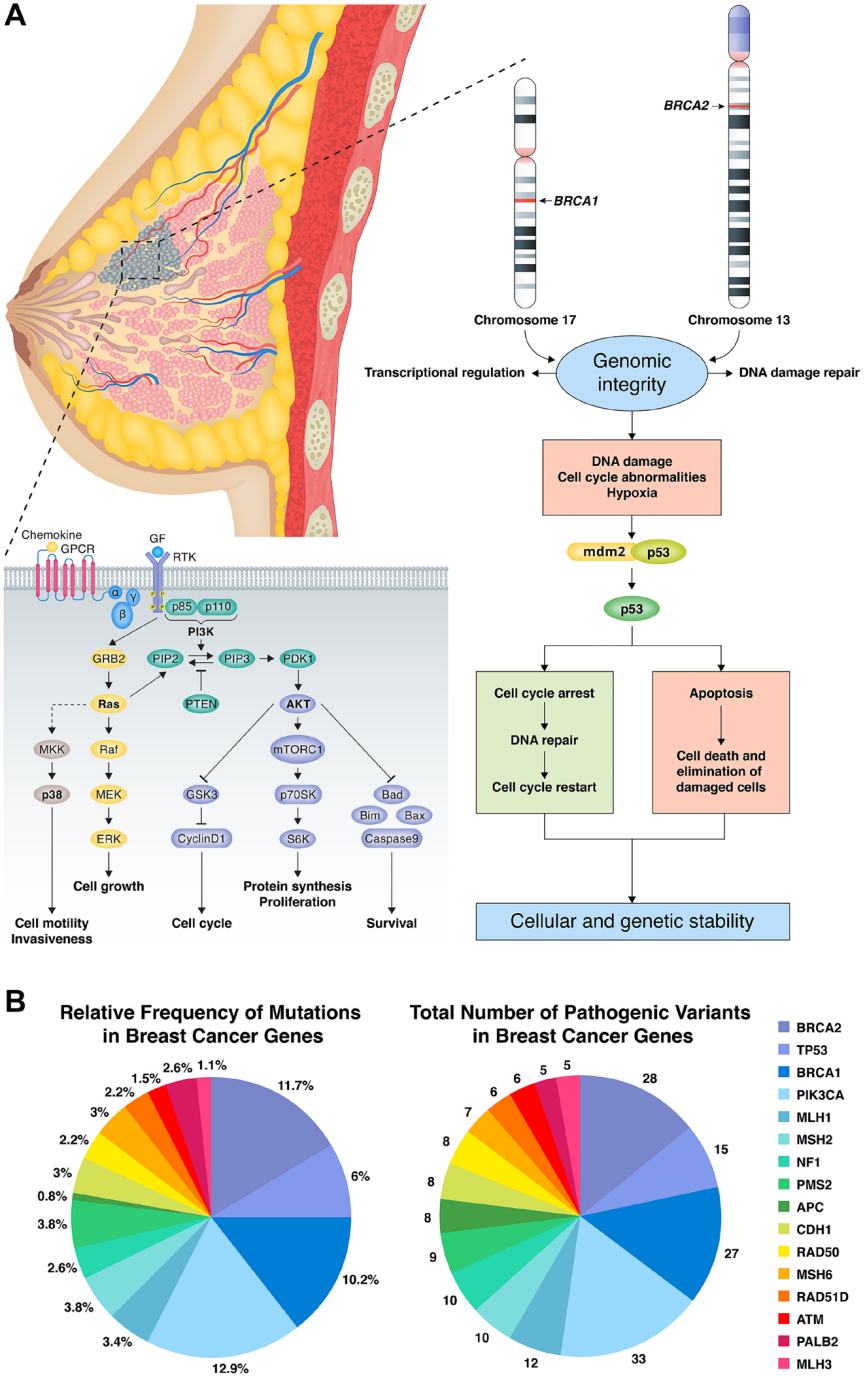
(**A**) BRCA1, BRCA2 and TP53 in DNA damage repair pathway resulting in cellular and genetic instability with potential points for targeted therapy. PIK3CA cellular pathway effects on cell cycle, invasiveness and survival with potential points for targeted therapy. (**B**) Number and percent of mutations for genes of interest. The most frequently mutated somatic genes were PIK3CA (12.9%), BRCA2 (11.7%), BRCA1 (10.2%), TP53 (6.0%), MSH2 (3.8%), PMS2 (3.8%), BARD1 (3.8%), MLH1 (3.4%), CDH1 (3.0%), RAD50 (3.0%), MSH6 (3.0%), NF1 (2.6%), RAD51D (2.2%), ATM (1.5%), PALB2 (2.6%), and MLH3 (1.1%).

Because the validation establishes the reproducible limit of detection at 10% allele fraction at 50× coverage, our laboratory has set a minimum tumor content of 20% neoplastic cell nuclei based on histologic evaluation as a preanalytic criterion for sequencing. Heterozygous somatic variants in a diploid tumor population would be expected to be identified in specimens meeting this criterion. Using an in-house pipeline and tertiary analysis, 263 mutations spanning 51 genes were filtered. The most frequently mutated somatic genes were PIK3CA (12.9%), BRCA2 (11.7%), BRCA1 (10.2%), TP53 (6.0%), MSH2 (3.8%), PMS2 (3.8%), BARD1 (3.8%), MLH1 (3.4%), CDH1 (3.0%), RAD50 (3.0%), MSH6 (3.0%), NF1 (2.6%), RAD51D (2.2%), ATM (1.5%), PALB2 (2.6%), and MLH3 (1.1%) (Figure 1). The cohort also included common recurrent variants. Recurrent variants included H1047R in PIK3CA (2.6% of cases), N550H in BRCA1 (1.15% of cases), c.1461_1462delinsCA in BARD1 (70% of cases), and I2285V in BRCA2 (0.6% of cases). On the other hand, 56.6% of our patients harbored PIK3CA mutations, while BRCA2, BRCA1 and TP53 were mutated in 37.7%, 30.18% and 24.5%, respectively (pathogenic, nonpathogenic and novel) (Table 1).

### Known mutations

We identified 123 previously reported mutations spanning 44 genes in 53 tumor samples. The vast majority of pathogenic variants were found in PIK3CA (24 variants), TP53 (12 variants), BRCA2 (10 variants), and BRCA1 (14 variants). PIK3CA carried the most common recurrent mutation in our sample (p.H1047R). Other pathogenic PIK3CA variants included p.Q546R, p.R412Q, p.E1037K, p.N1044K, p.H1047L, p.M1043I, p.H1065Y, p.E545K, p.R38C, and c.1060-17C>A (Supplementary Table 2).

### Novel mutations

Most of the novel variants identified were in BRCA2 (9 variants), with additional variants in PIK3CA (4 variants), BRCA1 (3 variants), and TP53 (3 variants) (Supplementary Table 2).

### Association with clinical characteristics and subtypes

Associations between common gene mutations (TP53, PIK3CA, BRCA 2 and BRCA1) and clinical characteristics are delineated in Table 2. Mutations in PIK3CA, BRCA1 and BRCA2 showed no significant association with patient age except for TP53 (*p* = 0.004). TP53 mutations were associated with ER- and PR-negative status (*P* = 0.003), in addition to a prominent *in situ* component. BRCA1 (*P* = 0.029) and BRCA2 (*P* = 0.038) variants were also associated with DCIS. There was no relationship between mutations in PIK3CA, BRCA1 and BRCA2 and subtype. Only the mutation in TP53 was significantly associated with subtype (*p* = 0.003).

**Table 2:** The association of gene mutations with age, subtype and DCIS

	BRCA1		*P*	BRCA2		*P*	PIK3CA		*P*	TP53		*P*
	Mutant	WT		Mutant	WT		Mutant	WT		Mutant	WT	
	*n* = 15	*n* = 25		*n* = 17	*n* = 23		*n* = 22	*n* = 18		*n* = 9	*n* = 31	
HER2 enriched	3 (20%)	4 (16%)		3 (17.65%)	4 (17.39%)		4 (18.18%)	3 (16.66%)		1 (11.11%)	6 (19.35%)	
Subtype^†^			0.618			0.057			0.838			0.003
Luminal	6 (40%)	14 (56%)		5 (29.41%)	15 (65.22%)		12 (54.54%)	8 (44.44%)		1 (11.11%)	19 (61.29%)	
TNBC	6 (40%)	7 (28%)		9 (52.94%)	4 (17.39%)		6 (27.27%)	7 (38.88%)		7 (77.78%)	6 (19.35%)	
	*n* = 16	*n* = 36		*n* = 19	*n **=*** 33		*n* = 29	*n* = 23		*n* = 14	*n **=*** 38	
DCIS			0.029			0.038			0.232			0.197
Absent	9 (56.25%)	9 (25%)		10 (52.63%)	8 (24.24%)		8 (27.59%)	10 (43.48%)		7 (50%)	11 (28.95%)	
Present	7 (43.75%)	27 (75%)		9 (47.37%)	25 (75.76%)		21 (72.41%)	13 (56.52%)		7 (50%)	27 (71.05%)	
Age			0.132			0.693			0.686			0.004
< 50	4 (25%)	17 (47.22%)		7 (36.84%)	14 (42.42%)		11 (37.93%)	10 (43.48%)		1 (7.14%)	20 (52.63%)	
≥ 50	12 (75%)	19 (52.78%)		12 (63.16%)	19 (57.58%)		18 (62.07%)	13 (56.52%)		13 (92.86%)	18 (47.37%)	

†Subtype data is available for 40 patients only. *WT*: Wild Type.

### Statistical results

**Figure 2:**
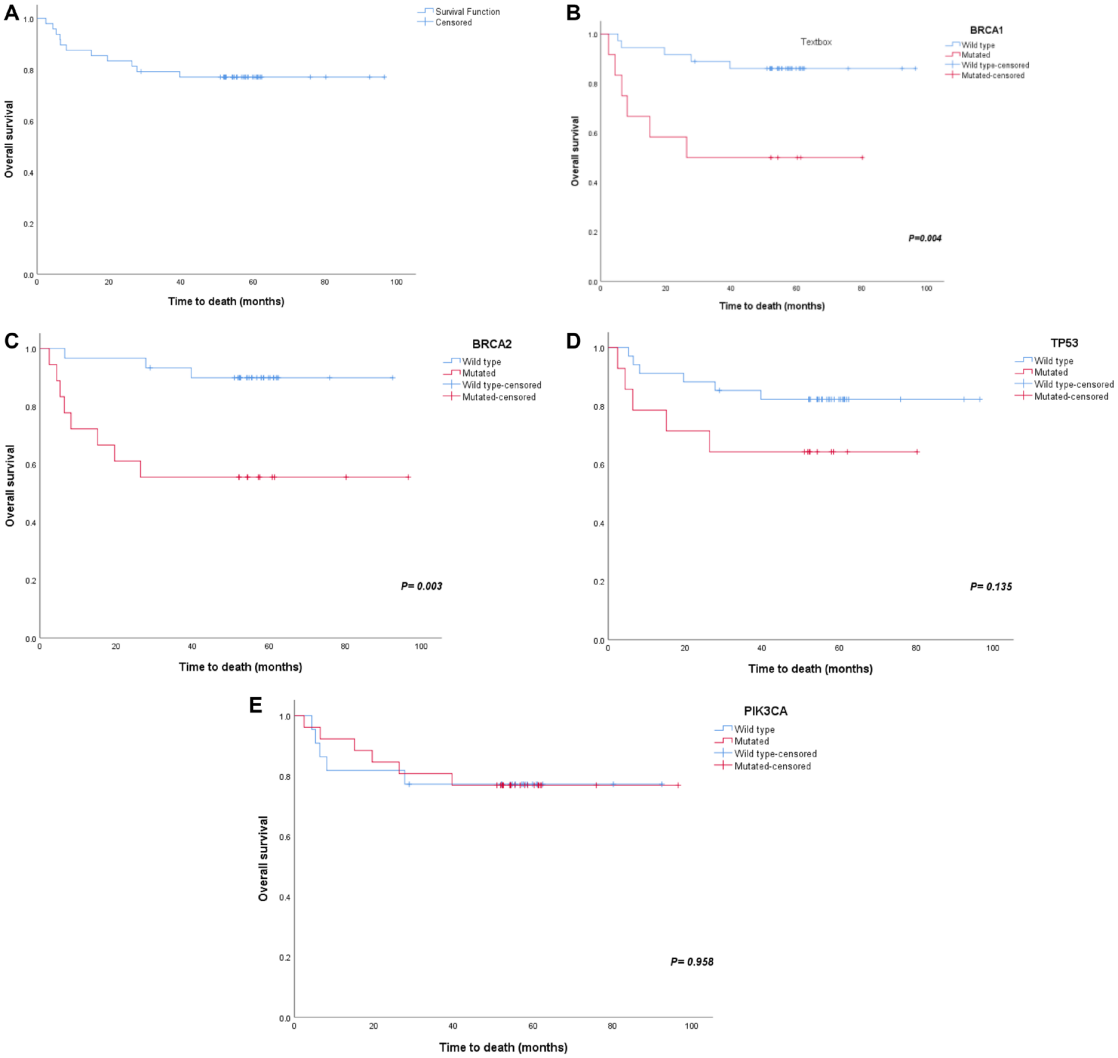
Survival plots for major gene mutations. Major gene mutations have showed effects on survival (**A**) overall survival, (**B**) BRCA1 survival, (**C**) BRCA2 survival, (**D**) TP53 survival and (**E**) PIK3CA survival.

The OS of our study patients was 77% (5-year overall survival) (Figure 2A). The survival probability was lower for patients with mutant BRCA1 (50% versus 86%); this difference was highly statistically significant (*p* = 0.004) (Figure 2B). Mutation in BRCA2 resulted in a lower survival rate as well (55.6% vs 89.9%; *p* = 0.003) (Figure 2C). A lower survival rate was observed with mutations in TP53 (64.3% versus 82.2%). However, there was no significant difference between the 2 rates (Figure 2D). There was no difference in survival between patients with and without PIK3CA mutations (76.9% vs 77.3%) (Figure 2E).

Univariate and multivariate Cox proportional hazards models were used to estimate the hazard ratios of carriers and noncarriers of the mutation (Table 3). Age was included as a prognostic factor in addition to gene mutations. Univariate analysis showed that patients with BRCA1 mutations had a 4.83-fold increased risk of dying (95% CI: 1.47–15.91, *P* = 0.010). Additionally, BRCA2 mutations had a 5.87-fold increased risk compared to that in noncarriers (95% CI: 1.55–22.21, *P* = 0.009). However, multivariate analysis showed BRCA2 to be the only independent factor that significantly contributed to decreased survival (HR = 5.14, 95% CI 1.16–22.80, *P* = 0.031).

**Table 3:** Univariate and multivariate cox proportional hazards model estimations for carriers of the mutation when compared to non-carriers

Factor	Univariate			Multivariate		
	HR	95% CI	*P*	HR	95% CI	*P*
**BRCA1**, mutated vs. wild type	4.83	1.47–15.91	0.010	3.26	0.65–16.38	0.152
**BRCA2**, mutated vs. wild type	5.87	1.55–22.21	0.009	5.14	1.16–22.80	0.031
**PIK3CA**, mutated vs. wild type	0.97	0.30–3.17	0.958	1.58	0.42–5.92	0.495
**TP53**, mutated vs. wild type	2.41	0.73–7.90	0.147	0.84	0.17–4.23	0.829
**Age**, ≥ 50 vs. < 50	1.79	0.48–6.77	0.388	1.12	0.25–5.01	0.881

## DISCUSSION

There is a high prevalence of BCa mortality among Saudi Arabian women, and the burden of BCa in the Arab world continues to grow [41, 42]. Despite its importance, our knowledge of the risk factors for BCa in Saudi and Arab women, in general, remains sparse [43]. The majority of Arab BCa cases are not caused by inherited genetic factors but are associated with somatic mutations in breast cells that accumulate over time [44]. In this study, to determine somatic mutation frequencies in Saudi Arabian women, we sequenced the protein-coding exons of 70 genes in tumor samples from 53 cases. A heavy mutation burden was detected in all BCa tumors. This is potentially a hallmark of increased genomic instability and may correlate with tumor aggressiveness. BRCA1, BRCA2, PIK3CA, and TP53 were the most frequently mutated genes, in agreement with previous studies [8, 45, 46]. PIK3CA was the most common somatic mutation in our cohort, as in other populations, with an additional prominence of TP53 (Figure 1), which aligns with the prevalence previously described in the region. In contrast, we did not detect high-frequency mutations of PTEN and RB1 as noted in other Arab cohorts [47, 48]. This may indicate distinct gene mutation frequencies in the Saudi BCa population.

The study cohort included common recurrent variants, including N550H in BRCA1 and H1047R in PIK3CA and P41L in BRCA2 [49–51]. Of the known pathogenic mutations, the majority were found in PIK3CA (24 variants), TP53 (12 variants), BRCA2 (10 variants), and BRCA1 (14 variants).

Genetic testing in BCa is rapidly advancing, and the ability to identify germline mutations in high-risk individuals permits increased surveillance and early genetic counseling. BRCA1 and BRCA2 germline mutations account for approximately 30% of heritable BCas globally and approximately 20% of Arab BCas [52–54]. In high-risk Saudi patients, BRCA1/BRCA2 mutation rates of 12.9% were reported in BCa tumors [55], while our cohort, on the other hand, had higher somatic mutations rates (30.18% for BRCA1 and 37.7% for BRCA2). Additionally, we report that patients with BRCA1 and BRCA2 mutations had shorter overall survival than patients without these mutations (*p* = 0.004 and *p* = 0.003, respectively) (Figure 2A and 2B). Cox hazard ratio analysis also showed a 4.83- and 5.87-fold increase in hazard ratio in samples with these mutations, while BRCA2 appeared to be the only independent factor contributing significantly to lower survival (Table 3).

BRCA1 and BRCA2 BCa have therapeutic relevance. As an example, PARP inhibitors are more effective in metastatic BCa patients with BRCA1/BRCA2 mutations than in those without these mutations. Olaparib (a PARP inhibitor) is now FDA-approved for treating metastatic BCa positive for BRCA1/BRCA2 mutations [56]. Further clinical trials investigating the use of cisplatin and olaparib as systemic therapies for BRCA-associated BCas are also underway and are showing favorable results [57–59]. The combination of these therapies with knowledge of lifestyle factors and other personal characteristics may further personalize treatments for BRCA1/BRCA2 BCas, revolutionizing treatment efficacy in the future.

PIK3CA carried the most common mutation across the samples (p.H1047R). Other potential pathogenic PIK3CA variants included p.Q546R, p.R412Q, p.E1037K, p.N1044K, p.H1047L, p.E545K, p.R38C, and c.1060-17C>A. Gain-of-function mutations in PIK3CA have been identified in many cancers with a global incidence of 26% and an incidence of approximately 29% in Arab BCas [48]. PIK3CA mutations are significantly associated with a lower BCa grade and hormone receptor positivity in Arab countries [48]. Our cohort did not show a significant difference with regard to these features or survival outcome. In comparison, a recent pooled analysis of ≥ 10,000 early-stage BCa patients with PIK3CA-mutated tumors showed an improved prognosis specific to ER, PR+/HER2– and TNBC subtypes, but not the HER2+ subtypes, which were associated with a reduced overall survival [1]. Our cohort included only four HER2+ subtypes, while the remainder of the subtypes included twelve luminal and six triple-negative BCas (Table 2), which reflects a similar behavior in those tumors. Additionally, PIK3CA was found in DCIS in 72.4% (Table 2), which affirms the reported literature of PIK3CA involvement in early breast carcinogenesis [45, 60]. From a treatment standpoint, the OncoKB and CIViC websites identified two actionable mutations in PIK3CA. The first was c.3140A>G, p.H1047R or p.H1047L, the most common pathogenic mutation which was altered in nine patients. According to CIViC, this mutation has an actionability score of 49 and should respond to alpelisib and fulvestrant, buparlisib and fulvestrant, fulvestrant and taselisib, alpelisib, buparlisib, copanlisib, GDC-0077, serabelisib and taselisib (OncoKB). The other mutation was c.1633G>A, p.E545K, which has an actionability score of 34. Genetic testing for PI3KCA mutations may, therefore, aid individualization of BCa treatment in Saudi women [61–63].

According to the literature, TP53 is mutated in approximately 80% of TNBC tumors [60]. Our cohort reported a 77.7% frequency of mutations in TNBC cancers (*p* = 0.003), which correlates with previously reported literature. Additionally, TP53 is reported to be associated with poor prognosis in triple-negative cancers [64]; however, our data did not show any significant difference when comparing the OS of carrier and noncarrier patients. Such patients can benefit from immune checkpoint inhibitors (ICIs), as patients with a mutant TP53 and wild-type PIK3CA demonstrate favorable immunotherapy-responsive signatures [65].

Taken together, we identified somatic mutation variants in Saudi BCa patients; BRCA1, BRCA2, TP53, and PIK3CA were found to be among the most common. In total, we identified 39 novel mutations that were not reported before and were predicted to be pathogenic. Our study has pertinent limitations. Further, our limited sample size, particularly for limited somatic genomics aberrations analyzes, may limit generalizability. However, we highlighted the importance of routine genetic sequencing in the implementation of precision therapies in Saudi Arabia. More regional studies are still needed.

## MATERIALS AND METHODS

Tissue samples were collected from 53 consenting Saudi patients in King Fahad Medical City (KFMC) diagnosed with BCa in the period of 2011–2015. All cases were followed up retrospectively for a median duration of 5 years. Cases were selected based upon cancer tissue content. ER/PR/HER2 immunohistochemical status was obtained from histopathology reports, all reported and published according to the ASCP/CAP guidelines (updated version of 2018). Samples were collected as FFPE (formalin-fixed, paraffin embedded) tumor blocks. FFPE tumors and hematoxylin-eosin-stained slides were prepared and reviewed by a pathologist (MA) to identify areas of ≥ 20% tumor for molecular analysis. The tumor-enriched areas were macrodissected or from ten 5-micron tissue sections or punched from the block. In order to obtain sufficient quantities of DNA, several isolations were performed from each sample, pooled to ensure homogeneity, and then aliquoted for use in validation. DNA was manually extracted from the blocks using the GeneRead DNA FFPE Kit (QIAGEN).

All samples underwent targeted sequencing using a customized panel designed by Thermo Fisher Scientific that has been verified for both sensitivity and specificity. The panel includes the following 70 known cancer genes: BRCA2, TP53, BRCA1, PIK3CA, MLH1, ATR, BARD1, ATM, PMS2, RAD50, NF1, MSH2, MSH6, RAD51D, ALK, CDH1, PRF1, RAD51C, APC, MLH3, MYH8, PALB2, PPM1D, PTEN, RET, BRIP1, AXIN2, CDC73, CDK12, CHEK2, EGFR, ELAC2, EXT2, FANCM, GPC3, HOXB13, KIT, PDGFRB, POLE, PTCH1, RB1, RECQL4, TSC1, VHL, AIP, AKT1, BAP1, BMPR1A, BUB1, CBL, DICER1, FANCI, HNF1A, INSRR, LIG4, MEN1, NF2, NTRK1, PALLD, PTCH2, RHBDF2, SETBP1, SMAD4, SMARCB1, SMARCE1, SPRED1, TMEM127, TSC2, WRN and WT1.

The panel was selected for its high coverage of genes related to BCa [66]. The samples were sequenced on the Ion GeneStudio S5 system. Data were generated from the Ion GeneStudio S5 system and underwent initial alignment and analysis by the SHGP pipelines. Pooled sample reads were deconvoluted and sorted using the Picard tools. Reads were aligned to the reference sequence b37 edition from the Human Genome Reference Consortium. Duplicate reads were identified and removed using Picard. Mutation analysis for single-nucleotide variants was performed using MuTect v. 1 0.2720 and annotated by Oncotator, developed by the Cancer Biology Group at the Broad Institute. For each sequencing run, nonneoplastic FFPE samples were included as controls. Variants identified in these control samples due to sequencing artifacts were filtered.

An average of 1,200 targeted sequencing variants was detected. Variants underwent extensive analysis to identify the single-nucleotide variants (SNVs) of interest. Variants were filtered to exclude those that occur at a populational frequency of greater than 0.1% in the Exome Sequencing Project database (http://evs.gs.washington.edu/EVS/). The VCF files generated by the system were used for gene and SNV analysis using a specific filtering pipeline as described before [66].

Specific focus was given to genes commonly implicated in BCa, including BRCA1, BRCA2, PIK3CA, TP53, and mismatch repair genes (MMR). Variants were classified into: previously reported variants (those in the Human Gene Mutation Database (HGMD) and/or the Catalogue of Somatic Mutations in Cancer (COSMIC)); novel variants, which are potential pathogenic variants that are not reported in any databases; and polymorphisms: variants not in HGMD or COSMIC or previously reported in the single nucleotide polymorphism database (dbSNP). Any filtered variants that were reported in COSMIC more than twice were rescued and presented for manual review.

We combined all SNVs identified in the selected genes and listed the genotype for each sample, removed duplicates (recurrent calls), and generated a list including the SNVs identified and the wild-type gene before assessing the association of these changes with selected clinical and pathological characteristics. A sample was considered wild type for a given gene if no mutations were found. Alterations have been previously assessed by mutation-specific PCR, pyrosequencing, or Sanger sequencing.

### Statistical analysis

Chi-square and Fisher’s exact tests were performed to examine the relationship between gene mutation and other variables, including age (above or below 50 years), BCa subtype designation (luminal, Her2 enriched, TNBC and unclassified) and DCIS (absent or present). Overall survival (OS) was analyzed by the Kaplan–Meier method; *p*-values were reported using the log-rank test. All cases selected for survival analysis were excisions, and hence, all have received the designated treatments and stages delineated. A Cox proportional hazards regression model was used to calculate the hazard ratios (HRs) and their 95% CIs for both univariate and multivariate models. All statistical analyses were performed using SPSS 25.0 software (SPSS Inc., Chicago, IL, USA); a *p*-value < 0.05 was considered statistically significant.

### Ethical approval and consent to participate

We declare that informed consent was obtained from all participants in adherence with the Declaration of Helsinki and the KFMC IRB and Research Advisory Committees (RAC) rules and regulations under the following approved project (KFMC IRB 16-310 MA). All protocols are carried out in accordance with relevant guidelines and regulations. All the methods are approved by the institutional and licensed Ethical Committee of KFMC IRB committee.

## SUPPLEMENTARY MATERIALS





## Abbreviations

BCa: Breast Cancer
GLOBOCAN: Global Cancer Incidence, Mortality and Prevalence No
IHC: Immunohistochemistry
RNA: Ribonucleic Acid
DNA: Deoxyribonucleic Acid
Ki-67: Marker of Proliferation
ER: Estrogen Receptor
PR: Progesterone Receptor
HER2: Human epidermal growth factor receptor 2
HDR: Homology-directed repair
Wnt: Wingless/Integrated
AT: Ataxia Telangiectasia
ATM: Ataxia Telangiectasia Mutated
ERE: Estrogen response elements
MAPK: Mitogen-activated protein kinase
PI3K: Phosphatidylinositol 4,5-bisphosphate 3-kinase
SHH: Sonic hedgehog
BRK: Breast tumor kinase
NGS: Next-generation sequencing
GWAS: Genome-wide association studies
WGA: Whole genome amplification
WES: Whole-exome sequencing
VEGF: Vascular endothelial growth factor
IGF: Insulin-like-growth-factor
PARP: Poly-ADP ribose polymerase
MMP: Matrix metalloproteases
SCR: Saudi Cancer Registry
ASR: Age-standardized rate
KFMC: King Fahad Medical City
ASCP: American society for clinical pathology
CAP: College of American Pathologists
FFPE: Formalin-fixed, paraffin embedded
SNVs: Single-nucleotide variants
VCF: Virtual Contact File
MMR: Mismatch repair genes
HGMD: Human Gene Mutation Database
COSMIC: Catalogue of Somatic Mutations in Cancer
dbSNP: Single nucleotide polymorphism database
PCR: Polymerase Chain Reaction
TNBC: Triple Negative Breast Cancer
DCIS: Ductal Carcinoma *In Situ*
HR: Hazard Ratios
OS: Overall survival
IDC: Invasive ductal carcinoma
BRCA1/2: Breast Cancer Gene 1/2
TP53: Tumor Protein 53
FDA: Food and drug administration
