# Supplementary material for: Landscape of somatic mutations in breast cancer: new opportunities for targeted therapies in Saudi Arabian patients
Source: Oncotarget. 2021 Mar 30;12(7):686–97. doi: 10.18632/oncotarget.27909 (PMC8021026; doi:10.18632/oncotarget.27909)
Supplement: Supplementary file 1 [file oncotarget-12-686-s001.pdf]

Landscape of somatic mutations in breast cancer: new opportunities for targeted therapies in Saudi Arabian patients

SUPPLEMENTARY MATERIALS

Supplementary Table 1: Individual patient clinical and pathological data

Age	Diagnosis	DCIS	Location	SBR Grade	Stage	ER	PR	HER2	Subtype designation
51	IDC	1	rt breast	III	IIA	0	0	1	HER2 Enriched
58	IDC	0	lt breast	II	IIA	0	0	1	HER2 Enriched
52	IDC	1	rt breast	III	IIA	0	0	0	TNBC
57	IDC	1	lt breast	III	IIIC	0	0	0	TNBC
55	IDC	1	lt breast	II	NS	1	0	0	Luminal
73	IDC	1	rt breast	II	IIIC	0	0	0	TNBC
56	IDC	0	lt breast	III	IIB	0	0	0	TNBC
58	IDC	1	rt breast	III	IIA	0	0	0	TNBC
74	IDC	0	lt breast	II	IIIB	NS	NS	NS	NS
58	IDC	1	rt breast	III	IIA	0	0	0	TNBC
61	IDC	1	rt breast	II	IIIA	1	1	1	HER2 Enriched
55	IDC	1	lt breast	II	IIA	1	1	0	Luminal
54	IDC	1	lt breast	II	IB	1	1	0	Luminal
53	IDC	1	rt breast	III	IIIC	NS	NS	NS	NS
52	IDC	0	rt breast	III	biopsy	1	1	0	Luminal
57	IDC	1	lt breast	III	IIIC	1	1	0	Luminal
68	IDC	0	lt breast	III	IIIB	0	0	1	HER2 Enriched
63	IDC	1	lt breast	II	IIB	1	1	0	Luminal
51	IDC	0	rt breast	NS	IIIB	0	0	0	TNBC
70	IDC	0	lt breast	II	IIA	1	1	0	Luminal
60	IDC	0	lt breast	II	IB	NS	NS	NS	NS
58	IDC	0	rt breast	III	IB	NS	NS	NS	NS
66	IDC	1	lt breast	I	IIA	NS	NS	NS	NS
61	IDC	1	lt breast	NS	IV	1	1	0	Luminal
55	IDC	0	rt breast	III	IIIC	0	0	0	TNBC
60	IDC	0	lt breast	II	IB	NS	NS	NS	NS
54	IDC	1	rt breast	II	IIA	1	1	1	Luminal
52	IDC	1	lt breast	II	IIA	1	1	0	Luminal
57	IDC	1	lt breast	II	IIB	NS	NS	NS	NS
45	IDC	1	rt breast	II	IIIA	1	1	0	Luminal
50	IDC	1	lt breast	II	IIA	NS	NS	NS	NS

50	IDC	1	rt breast	III	IIB	0	0	0	TNBC
38	IDC	1	rt breast	III	IIB	0	0	0	TNBC
48	IDC	1	lt breast	II	IV	NS	NS	NS	NS
38	IDC	1	rt breast	III	IIB	0	0	0	TNBC
46	IDC	1	rt breast	III	IIIB	1	1	0	Luminal
36	IDC	1	lt breast	II	IV	1	1	1	HER2 Enriched
43	IDC with atypical medullary cancer features	1	lt breast	II	IB	1	1	0	Luminal
44	IDC	1	lt breast	II	IIA	1	1	0	Luminal
40	IDC with micropapillary features	1	lt breast	NS	IIB	NS	NS	NS	NS
49	IDC	0	lt breast	II	biopsy	1	1	0	Luminal
49	IDC	0	lt breast	II	biopsy	1	1	0	Luminal
30	IDC	0	lt breast	III	biopsy	0	0	1	HER2 Enriched
46	IDC	1	rt breast	III	IV	1	1	1	HER2 Enriched
44	IDC	0	rt breast	III	IIB	0	0	0	TNBC
45	IDC	0	rt breast	I	IB	1	1	0	Luminal
43	IDC	1	lt breast	II	IIA	1	1	0	Luminal
35	IDC	0	rt breast	III	IIB	0	0	0	TNBC
48	IDC	1	lt breast	III	IIA	1	1	0	Luminal
48	IDC	1	rt breast	III	IIIC	1	1	0	Luminal
49	IDC	1	lt breast	II	IIB	NS	NS	NS	NS
36	IDC	0	rt breast	III	IIB	NS	NS	NS	NS
38	IDC	NS	NS	NS	NS	NS	NS	NS	NS

Scarff-Bloom-Richardson grade (SBR). estrogen Receptor (ER). progesterone Receptor (PR). human epidermal growth factor receptor 2(HER2). Invasive ductal carcinoma (IDC), invasive lobular carcinoma (ILC), ductal carcinoma in situ (DCIS), rt breast (right breast), lt breast (left breast). triple negative breast cancer (TNBC). IA (breast tumor that is less or equal to 2 cm with no axillary lymph node metastasis), IB (tumor in breast that is less than 2 cm with NS without axillary lymph node micro-metastasis – N1mi – less than 2 mm), IIA (either: tumor less than 2 cm with metastasis of 1 to 3 axillary lymph nodes metastasis – N1 – or tumor more than 2 cm and less than 5 cm with no lymph node metastasis), IIB (either: tumor more than 2 cm and less than 5 cm with metastasis of 1 to 3 lymph nodes metastasis – N1 – or tumor more than 5 cm with no lymph node metastasis), IIIA (either: tumor less than 5 cm with metastasis of 4 to 9 axillary lymph nodes – N2 – or tumor more than 2 cm with metastasis of 1 to 3 lymph nodes), IIIB (extension to chest and NS or skin, with NS without lymph node metastasis N1-N2), IIIC (any tumor size with metastasis of more than 10 axillary lymph nodes – N3), IV (detectable distant breast cancer metastasis with any tumor size with NS without lymph node metastasis N1-N3), BX (specimen is biopsy only, not yet staged) NS: not specified.

Supplementary Table 2: The associations of gene mutations with age, subtype and DCIS. See Supplementary Table 2
